# Risk factors for length of stay and charge per day differ between older and younger hospitalized patients with AML

**DOI:** 10.1002/cam4.1492

**Published:** 2018-04-16

**Authors:** Anita J. Kumar, Tobi Henzer, Angie Mae Rodday, Susan K. Parsons

**Affiliations:** ^1^ Institute for Clinical Research and Health Policy Studies Tufts Medical Center Boston Massachusetts; ^2^ Division of Hematology/Oncology Tufts Medical Center Boston Massachusetts

**Keywords:** Acute myeloid leukemia, charge per day, chemotherapy, hospitalization, length of stay

## Abstract

Acute myeloid leukemia (AML) is associated with frequent hospitalizations. We evaluated factors associated with length of stay (LOS) and charge per day (CPD) for admissions in older (≥60 years) and younger patients (<60 years). We identified patients with ICD‐9‐CM codes for AML or myeloid sarcoma in the 2012 HCUP‐NIS. In separate models based on age, we examined patient (sex, race, income, insurance payer, chronic conditions, chemotherapy administration, death) and hospital (type, geography) characteristics. Multivariable negative binomial regression estimated factor effects on LOS and CPD using rate ratios, with HCUP‐NIS weights. In 43,820 discharges, LOS was longer in patients <60 than ≥60 (6.8 vs. 5.4 days). For patients <60, longer LOS was seen with more chronic conditions (RR = 1.10), Black race (RR = 1.16), chemotherapy (RR = 2.27), and geography; shorter LOS was associated with older age (RR = 0.93), Medicare (RR = 0.83), and hospital type. For patients ≥60, longer LOS associated with chronic conditions (RR = 1.07) and Asian race (RR = 1.33). Shorter LOS associated with older age (RR = 0.86), higher income (RR = 0.93), and hospital type. For patients <60, higher CPD associated with chronic conditions (RR = 1.05), death (RR = 1.93), and geography; lower CPD associated with increasing age (RR = 0.96), Medicaid (RR = 0.93), and rural hospitals (RR = 0.65). For patients ≥60, higher CPD associated with Medicare (RR = 1.05), more chronic conditions (RR = 1.02), younger age (RR = 1.1), west geography (RR = 1.37), death (RR = 1.45), and Hispanic race (RR = 1.15). We identify predictors for increased healthcare utilization in hospitalized patients with AML, which differ within age groups. Future efforts are needed to link utilization outcomes with clinical treatments and response.

## Introduction

Acute myeloid leukemia (AML) is an aggressive hematologic malignancy characterized by clonal proliferation of myeloid precursors. AML is associated with morbidity and mortality due to both the disease and complications from treatment. Primary sequelae include those related to bone marrow failure and cytopenias (e.g., bleeding, anemia, infection) and those resulting from circulating myeloid blasts (e.g., leukostasis, tumor lysis syndrome). Initial treatment may include inpatient intensive “induction” cytotoxic chemotherapy, outpatient less intensive therapy with hypomethylating agents, supportive care (including low‐dose cytarabine), and—in a small number of patients—therapy on an investigational trial 1, 2, 3, 4. A small proportion of patients with high risk or relapsed disease undergo hematopoietic cell transplant (HCT). Patients receiving induction chemotherapy may be hospitalized for 4–6 weeks to manage complications arising from cytopenias. Patients receiving outpatient treatments may require hospitalization to manage infections and toxicities from cytopenias or actual treatment.

Due to the morbidity of AML, patients are frequently hospitalized for several potential reasons: new diagnosis requiring emergent diagnostic evaluation and management of acute illness; induction chemotherapy; toxicities from outpatient therapy; HCT or readmissions for post‐HCT toxicities; and supportive care and hospice. Inpatient care comprises a large percentage of the cost of caring for patients with AML 5, but there are scant data about nationwide patterns of inpatient hospitalizations for different patient populations and hospitals.

Variations in practice may affect length of stay (LOS) and charges for care, particularly in younger versus older patients (≥60 years). Older patients often present with higher‐risk disease, are more sensitive to the toxicity of chemotherapy, and hence are less likely to receive intensive induction chemotherapy and HCT 6, 7, 8. Their patterns of hospitalization may differ from younger patients, and, as the median age of AML diagnosis is 65 years, the older population is of particular interest 9. We evaluated variations in LOS and charge per day (CPD) based on patient and hospital characteristics, in younger and older patients hospitalized with AML.

## Methods

### Database

This study uses the 2012 Agency for Healthcare Research and Quality's Healthcare Cost and Utilization Project National Inpatient Sample (HCUP‐NIS). HCUP‐NIS is a 20% sample of all discharges from US hospitals, where the unit of analysis is the discharge rather than the patient. Data elements include patient demographics, comorbidities, hospital characteristics, healthcare utilization information, and ICD‐9‐CM diagnoses and procedure codes. Discharges from rehabilitation and long‐term acute care hospitals are excluded.

HCUP‐NIS provides weights for each discharge to produce nationally representative estimates. For example, a discharge in the HCUP‐NIS with a weight of five represents five discharges nationally. The 2012 HCUP‐NIS contains 7,296,968 discharges, representing 36,484,846 weighted discharges.

### Patient selection

We identified hospitalizations for AML using ICD‐9‐CM diagnosis codes for AML (205.00, 205.02) and myeloid sarcoma (205.30, 205.32) without remission or in relapse. We excluded AML and myeloid sarcoma in remission (205.01, 205.31) as to not capture planned, uncomplicated admissions for consolidative chemotherapy. To be included, discharges had to document an AML ICD‐9‐CM code and patients had to be ≥15 years old (weighted *n* = 52,895; unweighted *n* = 10,579). We chose 15 years as the minimum age cutoff in order to capture adolescents and young adults who may be treated by adult oncologists 10. From our total sample, we excluded 9075 weighted (1815 unweighted) discharges that had missing data in our variables of interest or were in outlier race and insurance categories (e.g., labeled “other”).

### Study design and outcomes

This retrospective cohort study evaluated factors associated with coprimary outcomes of LOS and CPD for AML hospitalizations. We included patient and hospital characteristics: age, sex, race/ethnicity, income, insurance payer, number of chronic conditions, death during admission, chemotherapy administration during admission, hospital type, and geographic region. We defined age as <60 years or ≥60 years to evaluate variations in associations between outcomes and covariates for younger versus older patients. This cutoff was based on a large study that identified age ≥60 years as a prognostic factor 11, and many clinical trials using ≥60 years as inclusion criteria for older patients with AML 2, 12, 13, 14, 15. HCUP‐NIS estimates income quartile using median household income for the patient's ZIP code. We collapsed the upper three quartiles representing higher income into one category, as modified from prior studies 16. HCUP‐NIS uses ICD.9 codes to identify chronic conditions, which are defined as those that last at least 12 months, and that place limitations on self‐care, independent living, and social interactions, or result in need for ongoing intervention 17. Hospital type is defined as urban teaching, urban nonteaching, or rural. Geographic region is defined as northeast, Midwest, south, or west, based on the US Census Bureau.

HCUP‐NIS determines LOS by subtracting the admission from the discharge date, giving the number of nights in the hospital. Patients discharged on the same day as admission incur a LOS of 0 nights. To calculate the CPD for each discharge, we divided total charges by LOS plus one.

### Statistical analysis

In separate models for younger (<60 years) and older (≥60 years) patients, we calculated summary statistics for all variables by age and compared them using chi‐square tests or linear regression. These analyses omitted discharges with missing data. Also using chi‐squared tests or linear regression, we compared sex, hospital type, geographic region, and patient age between discharges with complete data and discharges excluded due to missing data. Univariate and multivariable analyses were completed for all covariates by age group. All patient and hospital variables were included in the multivariable models to understand the effect of each variable while adjusting for the others. Given that the outcomes of LOS and CPD were skewed with small values for some hospitalizations (e.g., low CPD or short LOS), we planned to estimate multivariable models using Poisson regression. However, we found that the Poisson regression assumption of variance equaling the mean was not met (i.e., the data showed overdispersion), so negative binomial regression models were estimated instead. Rate ratios (RR) were calculated to represent increase or decrease in percent association with LOS and CPD. For example, a RR of 1.05 for LOS represents a 5% increase in LOS for a given variable compared to the reference. To understand whether the effect of hospital type varied by region, we explored the inclusion of an interaction term between these two factors in each multivariable model; results were reported if *P* < 0.05 for the interaction. HCUP‐NIS weights, strata, and clustering were applied to all analyses using either the survey procedures in SAS EG version 7.1 (SAS Institute, Inc., Cary, NC) for descriptive analyses and in Stata version 13 (College Station, TX: StataCorp LP) for negative binomial models. We report standard errors (SE) according to HCUP‐NIS guidelines 18.

## Results

### Patient and hospital characteristics

We identified 43,820 weighted discharges with complete data. Of these, 43,250 (98.7%) were AML, 280 (0.64%) were myeloid sarcoma, and 290 (0.66%) had both AML and myeloid sarcoma. Median age was 64.2 years (25–75%: 51.6–73.7) (Table 1). Most admissions (78.0%) were in White patients. Younger patients predominantly had private insurance (63.6%), while older patients primarily had Medicare (77.6%, *P* < 0.001). Most discharges were at urban teaching hospitals (73.2%), regardless of geographic region, (northeast: 84%, Midwest: 75%, south: 73%, west: 61%). The largest proportion of discharges was in the south (41.0%). Younger patients received inpatient chemotherapy more frequently than older patients (70.1% vs. 50.9%, *P* < 0.001).

**Table 1:** Patient and hospital characteristics

	Overall (*n* = 43,820 discharges)	Age 15–59 years (*n* = 16,655)	Age ≥60 years (*n* = 27,165)	*P*
Age (years), median (25–75%)	64.2 (51.6, 73.7)	46.7 (34.5, 53.8)	71.3 (65.8, 78.1)	–
Sex, % (SE)				
Male	55.2 (0.7)	52.4 (1.1)	57.0 (0.9)	<0.001
Female	44.8 (0.7)	47.6 (1.1)	43.0 (0.9)	
Race/Ethnicity, % (SE)				
Asian or Pacific Islander	2.8 (0.3)	3.3 (0.4)	2.5 (0.3)	<0.001
Black	11.3 (1.2)	14.5 (1.6)	9.3 (1.0)	
Hispanic	8.0 (0.6)	11.5 (1.1)	5.8 (0.5)	
White	78.0 (1.3)	70.7 (1.9)	82.4 (1.1)	
Income Quartile, % (SE)				
Upper 75%	75.1 (1.2)	73.6 (1.7)	76.0 (1.0)	0.07
Lower 25%	24.9 (1.2)	26.4 (1.7)	24.0 (1.0)	
Insurance, % (SE)				
Medicare	52.5 (0.9)	11.5 (0.7)	77.6 (0.7)	<0.001
Medicaid	11.1 (0.6)	24.8 (1.4)	2.6 (0.3)	
Private	36.5 (0.9)	63.6 (1.5)	19.8 (0.8)	
Number Chronic Conditions, median (25–75%)	5.6 (3.7, 7.6)	4.7 (3.0, 6.6)	6.0 (4.3, 8.1)	<0.001
Died during admission, % (SE)				
Yes	10.8 (0.3)	7.0 (0.5)	13.2 (0.5)	<0.001
No	89.2 (0.3)	93.0 (0.5)	86.8 (0.5)	
Chemotherapy, % (SE)				
Yes	37.9 (1.1)	49.1 (1.2)	29.9 (1.3)	<0.001
No	62.1 (1.1)	50.9 (1.2)	70.1 (1.3)	
Hospital type, % (SE)				
Urban nonteaching	21.8 (1.4)	13.7 (1.3)	26.7 (1.6)	<0.001
Rural	5.0 (0.4)	3.0 (0.5)	6.3 (0.5)	
Urban teaching	73.2 (1.7)	83.3 (1.5)	67.0 (1.9)	
Region, % (SE)				
West	18.0 (1.6)	19.9 (2.3)	16.9 (1.4)	0.01
Northeast	20.5 (2.0)	18.2 (2.4)	22.0 (1.8)	
Midwest	20.4 (2.1)	19.3 (2.7)	21.1 (1.8)	
South	41.0 (3.3)	42.5 (4.1)	40.1 (2.9)	

We found no differences in missing data by patient sex, hospital type, or region. Patients with complete data were 4.2 years older on average than those with missing data (*P* < 0.001).

### Length of stay

Median LOS was 5.8 days (25–75%: 3.1–18.0). LOS was longer in patients <60 years (6.8 days, 25–75%: 3.9–25.0) compared to patients ≥60 years (5.4 days, 25–75%: 2.7–13.2; *P* < 0.001). In multivariable analysis with no interactions, for patients <60 years, the following factors were associated with shorter LOS: older age (RR = 0.93 per 5 years within the age group, *P* < 0.001); Medicare (RR = 0.83, *P* < 0.001) compared to private; and urban nonteaching (RR = 0.75, *P* < 0.001) and rural hospitals (RR = 0.60, *P* < 0.001) compared to urban teaching hospitals (Table 2). In contrast, more chronic conditions (RR = 1.10, *P* < 0.001), chemotherapy (RR = 2.27, *P* < 0.001), and Black race (RR = 1.16, *P* = 0.01), and west and northeast region hospitals (compared to south) had longer LOS (west: RR = 1.16, *P* = 0.01, northeast: RR = 1.15, *P* = 0.01). We found an interaction between hospital type and region (*P* < 0.001) for patients <60 years. In the interaction model, rural hospitals in the south and Midwest had shorter LOS than urban teaching hospitals. In the northeast, Midwest, and west, urban nonteaching hospitals had short LOS than urban teaching hospitals (Table 3).

**Table 2:** Negative binomial regression model for length of stay

Variable	Univariate				Multivariable			
	<60 years		≥60 years		<60 years		≥60 years	
	Rate ratio (95% CI)	*P*	Rate ratio (95% CI)	*P*	Rate ratio (95% CI)	*P*	Rate ratio (95% CI)	*P*
Age (per 5 years)	0.96 (0.93, 0.98)	0.002	0.71 (0.69, 0.74)	<0.001	0.93 (0.90, 0.95)	<0.001	0.86 (0.83, 0.90)	<0.001
Sex								
Female	1.01 (0.94, 1.09)	0.84	1.00 (0.94, 1.07)	0.95	1.01 (0.94, 1.08)	0.78	1.04 (0.99, 1.10)	0.10
Male (ref)								
Income quartile								
Upper 75%	0.96 (0.87, 1.05)	0.38	0.97 (0.89, 1.05)	0.45	0.95 (0.86, 1.06)	0.36	0.93 (0.88, 1.00)	0.04
Lower 25% (ref)								
Insurance		<0.001		<0.001		0.002		0.53
Medicaid	1.02 (0.93, 1.12)	0.73	0.99 (0.80, 1.22)	0.91	0.92 (0.84, 1.01)	0.08	1.05 (0.88, 1.25)	0.63
Medicare	0.81 (0.73, 0.91)	<0.001	0.76 (0.70, 0.82)	<0.001	0.83 (0.74, 0.92)	<0.001	0.97 (0.90, 1.05)	0.41
Private (ref)								
Number of Chronic Conditions	1.12 (1.10, 1.13)	<0.001	1.08 (1.07, 1.09)	<0.001	1.10 (1.08, 1.12)	<0.001	1.07 (1.06, 1.08)	<0.001
Died								
Yes	1.12 (0.94, 1.32)	0.20	1.10 (1.01, 1.21)	0.03	1.11 (0.94, 1.30)	0.21	1.11 (1.03, 1.20)	0.01
No (ref)								
Race/Ethnicity		0.12		0.006		0.03		0.002
Asian or Pacific Islander	1.11 (0.89, 1.39)	0.37	1.12 (0.93, 1.35)	0.21	1.04 (0.87, 1.24)	0.68	1.33 (1.12, 1.58)	<0.001
Black	1.09 (0.98, 1.21)	0.11	1.20 (1.07, 1.34)	0.002	1.16 (1.04, 1.29)	0.01	1.14 (1.01, 1.29)	0.03
Hispanic	1.11 (0.99, 1.25)	0.08	1.11 (0.96, 1.27)	0.16	1.12 (0.99, 1.28)	0.08	1.06 (0.95, 1.18)	0.31
White (ref)								
Chemotherapy								
Yes	2.5 (2.26, 2.77)	<0.001	3.12 (2.91, 3.34)	<0.001	2.27 (2.07, 2.49)	<0.001	2.75 (2.56, 2.96)	<0.001
No (ref)								
Hospital type		<0.001		<0.001		<0.001		<0.001
Urban nonteaching	0.62 (0.55, 0.70)	<0.001	0.62 (0.57, 0.68)	<0.001	0.75 (0.67, 0.85)	<0.001	0.85 (0.79, 0.91)	<0.001
Rural	0.36 (0.27, 0.49)	<0.001	0.45 (0.40, 0.51)	<0.001	0.60 (0.47, 0.76)	<0.001	0.77 (0.69, 0.86)	<0.001
Urban teaching (ref)								
Region		0.004		0.40		0.003		0.16
West	1.20 (1.05, 1.37)	0.008	0.90 (0.78, 1.02)	0.11	1.16 (1.03, 1.31)	0.01	0.92 (0.84, 1.02)	0.12
Northeast	1.18 (1.06, 1.31)	0.002	0.99 (0.86, 1.13)	0.84	1.15 (1.04, 1.27)	0.01	1.01 (0.93, 1.09)	0.86
Midwest	1.10 (0.98, 1.23)	0.09	0.99 (0.85, 1.15)	0.89	1.00 (0.92, 1.10)	0.96	0.94 (0.86, 1.02)	0.14
South (ref)								

**Table 3:** LOS and CPD with interaction between hospital type and geographic region

Variable	LOS				Charge per day			
	<60 years		≥60 years^a^		<60 years^a^		≥60 years	
	Rate ratio (95% CI)	*P*	Rate ratio (95% CI)	*P*	Rate ratio (95% CI)	*P*	Rate ratio (95% CI)	*P*
South								
Urban Teaching (ref)								
Rural	0.50 (0.36, 0.68)	<0.001	‐‐‐	‐‐‐	‐‐‐	‐‐‐	0.77 (0.66, 0.89)	0.001
Urban Nonteaching	0.88 (0.75, 1.03)	0.10	‐‐‐	‐‐‐	‐‐‐	‐‐‐	1.02 (0.90, 1.15)	0.75
Northeast								
Urban Teaching (ref)								
Rural	0.92 (0.63, 1.34)	0.68	‐‐‐	‐‐‐	‐‐‐	‐‐‐	0.52 (0.39, 0.69)	<0.001
Urban Nonteaching	0.32 (0.23, 0.43)	<0.001	‐‐‐	‐‐‐	‐‐‐	‐‐‐	0.79 (0.68, 0.92)	0.003
Midwest								
Urban Teaching (ref)								
Rural	0.37 (0.28, 0.48)	<0.001	‐‐‐	‐‐‐	‐‐‐	‐‐‐	0.66 (0.58, 0.75)	<0.001
Urban Nonteaching	0.57 (0.44, 0.74)	<0.001	‐‐‐	‐‐‐	‐‐‐	‐‐‐	0.86 (0.77, 0.96)	0.01
West								
Urban Teaching (ref)								
Rural	0.71 (0.42, 1.20)	0.20	‐‐‐	‐‐‐	‐‐‐	‐‐‐	0.60 (0.52, 0.68)	<0.001
Urban Nonteaching	0.76 (0.61, 0.94)	0.01	‐‐‐	‐‐‐	‐‐‐	‐‐‐	1.10 (0.98, 1.22)	0.11

a Interactions between hospital type and geographic region were not significant for LOS ≥60 years or CPD <60 years, so results are not reported.

For patients ≥60, multivariable analysis demonstrated that older age (RR = 0.86 per 5 years, *P* < 0.001), higher income (RR = 0.93, *P* = 0.04), and urban nonteaching (RR = 0.85 *P* < 0.001) and rural hospitals (RR = 0.77, *P* < 0.001) compared to urban teaching hospitals had shorter LOS. Chemotherapy (RR = 2.75, *P* < 0.001), more chronic conditions (RR = 1.07 *P* < 0.001), Asian/Pacific Islander (RR = 1.33, *P* < 0.001), and Black race/ethnicity (RR = 1.14, *P* = 0.03) compared to White were associated with longer LOS (Table 2). There was no interaction between hospital type and geographic region in this age group.

### Total charge and charge per day

Median total charge per admission was $51,638 (25–75%: $23,262–147,292) for the entire cohort. Median CPD for the entire cohort was $6614 (25–75^%^: $4346–9794). CPD was higher in patients <60 years ($6836, 25–75^%^: $4525–10,418) than patients ≥60 years ($6492, 25–75^%^: $4255–9494, *P* < 0.001).

In multivariable regression, for patients <60 years, higher CPD was associated with more chronic conditions (RR = 1.05, *P* < 0.001), death during hospitalization (RR = 1.93, *P* < 0.001), Hispanic ethnicity (compared to White; RR = 1.14, *P* < 0.001), and west (RR = 1.47, *P* < 0.001) and northeast (RR = 1.27, *P* < 0.001) geography compared to south (Table 4). Lower CPD was associated with older age (RR = 0.96 per 5 years, *P* < 0.001), Medicaid insurance (compared to private; RR = 0.93, *P* = 0.03), and rural hospitals (compared to urban teaching; RR = 0.66, *P* < 0.001). There was no interaction between geography and hospital type (Table 3).

**Table 4:** Negative binomial regression model for charge per day

Variable	Univariate				Multivariable			
	<60 years		≥60 years		<60 years		≥60 years	
	Rate ratio (95% CI)	*P*	Rate ratio (95% CI)	*P*	Rate ratio (95% CI)	*P*	Rate ratio (95% CI)	*P*
Age (per 5 years)	0.98 (0.95, 1.00)	0.09	0.92 (0.90, 0.95)	<0.001	0.96 (0.93, 0.98)	<0.001	0.91 (0.89, 0.94)	<0.001
Sex								
Female	0.97 (0.91, 1.03)	0.32	0.97 (0.93, 1.00)	0.06	0.97 (0.92, 1.02)	0.29	0.97 (0.94, 1.00)	0.06
Male (ref)								
Income Quartile								
Upper 75%	1.13 (1.05, 1.22)	0.001	1.10 (1.03, 1.17)	0.002	1.05 (0.99, 1.11)	0.12	1.02 (0.96, 1.08)	0.50
Lower 25% (ref)								
Insurance		0.68		0.08		0.09		<0.001
Medicaid	0.99 (0.92, 1.07)	0.81	0.87 (0.76, 1.00)	0.04	0.93 (0.87, 0.99)	0.03	0.86 (0.77, 0.97)	0.01
Medicare	0.96 (0.89, 1.05)	0.38	0.95 (0.90, 1.00)	0.07	0.96 (0.89, 1.04)	0.30	1.05 (1.00, 1.11)	0.04
Private (ref)								
Number of Chronic Conditions	1.05 (1.03, 1.06)	<0.001	1.02 (1.01, 1.03)	<0.001	1.05 (1.03, 1.06)	<0.001	1.02 (1.01, 1.02)	<0.001
Died								
Yes	2.01 (1.79, 2.26)	<0.001	1.49 (1.41, 1.57)	<0.001	1.93 (1.73, 2.16)	<0.001	1.45 (1.37, 1.52)	<0.001
No (ref)								
Race/Ethnicity		0.02		<0.001		0.03		0.003
Asian or Pacific Islander	1.12 (0.92, 1.35)	0.25	1.11 (1.00, 1.23)	0.04	1.04 (0.89, 1.22)	0.62	0.98 (0.89, 1.07)	0.63
Black	0.97 (0.90, 1.04)	0.40	1.01 (0.93, 1.09)	0.88	1.03 (0.95, 1.11)	0.50	1.03 (0.95, 1.11)	0.46
Hispanic	1.21 (1.07, 1.37)	0.002	1.20 (1.11, 1.30)	<0.001	1.14 (1.04, 1.25)	<0.001	1.15 (1.07, 1.24)	<0.001
White (ref)								
Chemotherapy								
Yes	0.97 (0.91, 1.04)	0.43	1.10 (1.04, 1.16)	<0.001	0.95 (0.9, 1.00)	0.06	1.04 (0.99, 1.09)	0.10
No (ref)								
Hospital type		0.002		<0.001		<0.001		<0.001
Urban nonteaching	0.95 (0.85, 1.07)	0.38	0.98 (0.91, 1.06)	0.63	0.93 (0.84, 1.04)	0.24	0.97 (0.90, 1.04)	0.36
Rural	0.61 (0.46, 0.80)	<0.001	0.59 (0.53, 0.66)	<0.001	0.65 (0.52, 0.81)	<0.001	0.67 (0.61, 0.74)	<0.001
Urban teaching (ref)								
Region		<0.001		<0.001		<0.001		<0.001
West	1.46 (1.21, 1.76)	<0.001	1.40 (1.23, 1.59)	<0.001	1.47 (1.23, 1.76)	<0.001	1.37 (1.21, 1.55)	<0.001
Northeast	1.26 (1.07, 1.49)	0.005	1.13 (1.01, 1.26)	0.04	1.27 (1.08, 1.48)	<0.001	1.12 (1.00, 1.25)	0.05
Midwest	0.97 (0.85, 1.10)	0.66	0.96 (0.87, 1.07)	0.45	0.99 (0.88, 1.12)	0.93	0.95 (0.87, 1.04)	0.27
South (ref)								

For patients ≥60 years, multivariable regression with no interactions indicated that higher CPD was associated with Medicare (compared to private; RR = 1.05, *P* = 0.04), more chronic conditions (RR = 1.02, *P* < 0.001), death during hospitalization (RR = 1.45, *P* < 0.001), and Hispanic ethnicity (compared to White; RR 1.15, *P* < 0.001). Older age (RR = 0.91 per 5 years, *P* < 0.001), Medicaid (compared to private; RR = 0.86, *P* = 0.01), and rural hospitals (compared to urban teaching, RR = 0.67, *P* < 0.001) were associated with lower CPD. Hospitals in the west had higher CPD (RR = 1.37, *P* < 0.001) than in the south (Table 4). We found an interaction between hospital type and region (*P* < 0.001). In the interaction model, rural hospitals had lower CPD than urban teaching hospitals across all regions, but CPD in urban teaching and nonteaching hospitals varied by region. In the northeast and Midwest, urban nonteaching hospitals had lower CPD than urban teaching hospitals, while there was no difference in CPD by these two hospital types in the south or west (Table 3).

## Discussion

In a population database, we identified factors associated with LOS and CPD in patients hospitalized with AML, based on clinically relevant age groups. In a single year (2012), there were 43,820 weighted discharges in this rare disease. To put this into perspective, there will be an estimated 21,380 projected new cases in 2017 19, suggesting patients experience multiple admissions annually. As AML is primarily a disease of older age 9, our findings reveal that older patients have more chronic conditions, higher death rates, receipt of less chemotherapy, and are hospitalized less often in urban teaching hospitals compared to younger counterparts. This is noteworthy, as treatment in an academic center may portend better overall survival 20. We do not know whether referring physicians are less likely to transfer older patients to urban teaching hospitals because they believe patients will not derive benefit, but prior database analyses suggest this population may be undertreated, despite aggressive approaches leading to better survival 21, 22.

Median LOS in our cohort was 6 days, with the top 25% of admissions having LOS over 18 days. As inpatient induction chemotherapy typically requires 4–6 weeks of hospitalizations, our analysis suggests that induction chemotherapy admissions comprise a minority of AML admissions, further supported by the finding that <40% of admissions involved chemotherapy. We did not include admissions for AML in remission as to exclude planned consolidative chemotherapy admissions. Rather, the majority of admissions in our analysis were likely associated with new diagnoses or treatment complications. We also found that younger patients had longer LOS than older patients. At first glance, median LOS in younger patients was only 1.4 days longer. However, the upper quartile for LOS was >13 days for older patients and >25 days for younger patients, indicating more younger patients had longer LOS. This is consistent with older patients dying more frequently in the hospital, which we found, and fewer older patients receiving inpatient induction chemotherapy 7, 22.

More chronic conditions, chemotherapy, and admission to an urban teaching hospital (vs. rural) portended longer stays, suggesting that more aggressive care, or sicker patients are associated with longer LOS 23. Chronic conditions, urban teaching hospitals, and dying during hospitalization were associated with higher CPD. Palliative/end‐of‐life care is utilized less for hematologic malignancies than for solid tumor cancers, partly due to the challenge in identifying when disease is untreatable 5. Similarly, patients often become critically ill rapidly and unpredictably in hematologic malignancies, which is consistent with greater resource utilization at the end of life, particularly when requiring intensive care unit level care 24. Interventions to incorporate hospice for AML patients at the end of life may reduce costs. Interestingly, in our analysis, chemotherapy did not impact CPD for older patients, in contrast to prior studies of elderly patients 23. This may reflect the paucity of costly novel agents available in 2012 and the reliance on less expensive traditional chemotherapies. However, this finding also highlights that comorbidity may drive costs more than treatment choice in this population.

The role of geography in our analysis of LOS and CPD is less clear. We found an interaction between hospital type and geography only among younger patients’ LOS. This may not be present in older patients, if they are treated more uniformly with palliative regimens across different regions, and less likely to be referred to academic centers 23. In contrast, for CPD, we found an interaction between hospital type and geography only in older patients. Lowest CPD was seen across all geographies in rural hospitals, presumably due to less aggressive and costly interventions offered, but there was no difference in CPD between urban teaching and nonteaching hospitals in the south and west suggesting more uniform approaches in these settings.

Insurance and financial status may influence LOS and CPD. Older patients in the highest income quartile had shorter LOS than lower income patients. Younger patients had less variation by income status and may be less susceptible to income affecting support after discharge. We found shorter LOS in younger patients with Medicare insurance. As Medicare in younger patients is often due to long‐term disability, it is difficult to generalize reasons for shorter LOS. Future analysis may elucidate utilization patterns in this subgroup. In contrast, older patients with Medicare had higher CPD than those with private insurance. This may partly reflect differences in patients within this older cohort by age (60–64 vs. ≥65), comorbidity, and overall severity of illness. There may also be differences in which treatments were rendered within this cohort. As hypomethylating agents are more costly than standard induction 25, a higher CPD in Medicare patients may be due to this older subset of these patients receiving their first cycle of hypomethylating agent treatment in the hospital and thus incurring a higher CPD.

In all patients, Black race was associated with longer LOS, and in older patients, Asians/Pacific Islanders also had longer LOS. In both age groups, Hispanic patients had higher CPD. Understanding the implications of race/ethnicity on healthcare utilization is challenging in a population database. Prior studies have demonstrated that Black patients may have genetic predisposition for neutropenia, which may account for prolonged stays 26. However, there are likely socioeconomic factors that are not captured in this dataset that limit our ability to draw inferences.

The most significant limitation of our study was that HCUP‐NIS does not identify specific chemotherapy agents administered during hospitalization. Patients who received induction likely have longer LOS, but we could not identify these admissions, nor could we evaluate treatment responses for those patients who received chemotherapy. We were unable to accurately identify admissions that followed HCT or involved intensive care unit level care, both of which may impact LOS and CPD 24. Finally, HCUP‐NIS does not allow for linkage of multiple hospitalizations per patient, including which admissions were associated with a transfer from another hospital. Transfers to tertiary care centers are common for AML, and one transfer would split one admission into two shorter hospitalizations, thereby underestimating the full LOS for an individual.

As practice patterns evolve, we may see a shift in LOS. Recent research has evaluated the feasibility and safety of outpatient postinduction management, rather than prolonged hospitalization 27, 28, 29, 30, 31, 32. CPD may be lower in patients who are discharged early, with some complications managed in the outpatient setting 32. A shift toward more outpatient management may reduce the overall economic burden and inpatient healthcare utilization.

We identified risk factors for longer LOS and higher CPD in younger and older patients hospitalized with AML. Future studies are needed to understand how specific treatments impact healthcare utilization, particularly in the elderly population, in whom the decision for aggressive chemotherapy, novel therapeutics, or clinical trial is more complex than in younger patients. Understanding of risk factors that increase utilization may lead to practice changes to reduce the burden of AML on individual patients and the healthcare system.

## Conflict of Interest

The authors have no conflict of interest to disclose.
